# Parents’ Views on Engaging Families of Middle School Students in Obesity Prevention and Control in a Multiethnic Population

**DOI:** 10.5888/pcd11.130138

**Published:** 2014-04-03

**Authors:** Burton O. Cowgill, Paul. J. Chung, Lindsey R. Thompson, Jacinta Elijah, Sheila Lamb, Vanessa P. Garcia, Roshan Bastani

**Affiliations:** Author Affiliations: Paul. J. Chung, UCLA Fielding School of Public Health, Jonsson Comprehensive Cancer Center, UCLA/RAND Prevention Research Center, Mattel Children’s Hospital UCLA, David Geffen School of Medicine at UCLA, Los Angeles, California, The RAND Corporation, Santa Monica, California; Lindsey R. Thompson, Jacinta Elijah, Jonsson Comprehensive Cancer Center, UCLA/RAND Prevention Research Center, Los Angeles, California; Sheila Lamb, Los Angeles Unified School District, Los Angeles, California; Vanessa P. Garcia, UCLA/RAND Prevention Research Center, Mattel Children’s Hospital UCLA, David Geffen School of Medicine at UCLA, Los Angeles, California; Roshan Bastani, UCLA Fielding School of Public Health, Jonsson Comprehensive Cancer Center, UCLA/RAND Prevention Research Center, Los Angeles, California.

## Abstract

**Introduction:**

Overweight and obesity remain significant public health risks for youth in the United States, particularly among racial/ethnic minority groups. Efforts at obesity prevention and control have targeted youth and family members in diverse settings. Although involving parents in obesity prevention programs for youth may improve the potential of these programs, less is known about parents’ preferred methods of engagement, especially among racial/ethnic minority parents and parents whose primary language is not English. In this qualitative study, parents of middle-school–aged children were asked how best to engage their children in obesity prevention and control efforts.

**Methods:**

We recruited 38 parents whose children attended Los Angeles middle schools to participate in focus groups. Two English-language focus groups with 14 parents of different racial/ethnic backgrounds and 2 Spanish language groups with 24 Latino parents were conducted from 2010 through 2011. We analyzed focus group transcripts by using content analysis using inductive and deductive techniques.

**Results:**

Findings from focus groups confirmed that parents want to help their children avoid obesity but feel constrained in their ability to take action. Participants identified an overarching desire to become better parents as a potential incentive to engage in obesity prevention efforts. Parents advocated for family-focused approaches in obesity prevention programs, including family sports leagues and cooking classes. Most findings were consistent between language groups, but parents in the Spanish language groups cited language-related barriers.

**Conclusion:**

The development and testing of simple programs that are sustainable, community-based, and family-focused may empower families to address obesity prevention and control.

## Introduction

The prevalence of obesity in the United States has tripled over the past 30 years; 32% of children aged 2 to 19 years are overweight or obese (1). The proportion is even higher in Los Angeles, where 42% of middle-school–aged children are overweight or obese (2). Most Los Angeles youths are racial/ethnic minorities and exhibit high obesity rates; 46% of Latino youth and 39% of African American youth are overweight or obese compared with 27% of non-Hispanic white youth (2). The immediate health implications for obese youth include increased risk for high cholesterol, high blood pressure, and prediabetes (3); moreover, obese youth are at high risk of becoming obese adults, with all of the long-term health risks obesity entails.

Multiple settings, including schools, homes, and community-based organizations, have been used to address childhood obesity (4,5). Despite these efforts, interventions in these settings have yielded limited results (6,7); one possible reason is that parents are seldom fully integrated into these interventions. Involving parents in efforts to reduce childhood obesity may be an effective approach because parents influence their children’s eating behaviors and their physical activity behaviors (8–12). However, identifying ways to successfully involve parents remains a challenge.

Parental involvement in childhood obesity programs has usually been approached by including parents in child-focused interventions (parent-involved) or by engaging the entire family unit (family-focused) (9–11,13,14). Engaging non-English–speaking parents in obesity prevention, however, has challenged public health practitioners because of cultural barriers, competing family demands, and social role norms (15,16). Consequently, interventions aimed at incorporating the non-English-speaking population in obesity prevention efforts frequently have low participation rates and high attrition (15,17,18).

Our study included a multiethnic sample of parents of middle school students in Los Angeles. We conducted English- and Spanish-language focus groups to elicit parents’ views on how to effectively engage parents in childhood obesity prevention and control efforts. Our objective was to inform development of obesity prevention programs aimed at reaching families with middle-school–aged children (children aged 11–14 years).

## Methods

### Eligibility and recruitment

Our sample consisted of parents of children enrolled in middle schools in the southwestern region of Los Angeles, California. Recruitment was done in collaboration with community organizations by posting flyers at school-based parent centers and through direct recruitment at school events. Parents received information about the study’s purpose, required time commitment, and eligibility criteria.

Eligible participants were parents who spoke English or Spanish and had a child enrolled in a Los Angeles middle school (grades 6–8) during the 2010–2011 school year. Sixty-four parents volunteered, and 46 were eligible. The study received institutional review board approval from the University of California, Los Angeles (UCLA), and we obtained informed consent from participants in English and Spanish.

### Focus groups

Of 46 parents selected for the 4 focus groups, 38 participated. We conducted 2 English-language groups with 14 parents of different racial/ethnic backgrounds and 2 Spanish-language groups with 24 Latino parents who preferred to communicate in Spanish. The parents who participated in the Spanish-language focus groups are referred to as “Spanish-language parents” throughout the article. We stratified our focus groups by language because Los Angeles schools are over 70% Latino, and language served as a proxy for acculturation during recruitment. The overall racial/ethnic distribution of participants mirrored that of the Los Angeles Unified School District. Each focus group included 5 to 12 parents, a trained moderator, and a note taker. All sessions were digitally recorded and transcribed.

Experienced moderators conducted the 2-hour focus groups at community sites on weekend and weekday mornings. Focus groups were guided by a semistructured protocol based on a review of youth-focused obesity-prevention literature (Appendix) and the study team’s experience in delivering school-based obesity prevention interventions. Moderators encouraged parent interaction and open discussion of diverse views.

Parents completed a questionnaire to provide demographic information, household composition, and child and parent self-reported height and weight. The designations of overweight and obese for children and parents are based on the Centers for Disease Control and Prevention’s age–sex growth charts (19). Acculturation was assessed for Latino participants by using the Short Acculturation Scale for Hispanics (20). Parents reported data on their oldest middle-school–aged child. Participants received a $40 incentive.

### Qualitative analysis

Transcripts were imported into Atlas.ti (Scientific Software Development GmbH, Berlin, Germany). Proceedings of Spanish-language groups were transcribed into Spanish, translated into English for analysis, and independently verified for accuracy by a bilingual research assistant. Content analysis of the transcripts was conducted by using both inductive and deductive techniques (21). We created a set of theme-based codes, applied them systematically to the narratives, and tested reliability between coders (21). The first author (B. C.) reviewed the transcripts to identify major thematic content that either fit within categories included in the focus group protocol (ie, preferred program content and barriers and facilitators for parental engagement) or emerged spontaneously from the groups. Two coders with extensive experience in qualitative coding and in working with diverse populations on obesity prevention were given basic operational definitions of these themes and were instructed to identify all related text. One author (B. C.) resolved discrepancies between coders. This procedure resulted in 735 comments.

Although participants did discuss topics in the general protocol categories, subthemes were allowed to emerge spontaneously and often developed tangentially to the anticipated content. The coders identified these subthemes by sorting comments into categories both within and across the protocol categories (21). This process resulted in the identification of the 3 central themes and an overarching meta-theme that described parents’ motivations to participate in obesity prevention efforts. Cohen’s κ was used to check consistency between coders (22) and was satisfactory or better for the themes (0.72–0.89) (23). To compare English-language and Spanish-language focus groups, we analyzed comments by language and assessed similarities and differences between the groups to identify the role acculturation and English played in participant responses.

## Results

### Participant characteristics

Most of the 38 participants were women (97%) with a mean age of 42 years (Table). On the basis of self-reported height and weight, 47% of parents were obese and 31% were overweight. Parents in the Spanish-language group reported low acculturation (average 1.6 on 1–5 scale where 1 is least acculturated and 5 is most acculturated) compared with a mean of 3.3 for Latino parents in the English-language groups. The children parents discussed had a mean age of 12.1 years (range 11 y –14 y). On the basis of parents’ report of their child’s height and weight, 39% of children were obese and 11% were overweight.

**Table:** Parent and Child Characteristics, Parents’ Views on Engaging Middle School Students in Obesity Prevention and Control in a Multiethnic Population, Los Angeles, California, 2010–2011

Characteristic^a^	Spanish (n = 24)	English (n = 14)	Total (N = 38)
**Parents**			
**Sex, %**			
Male	0	7	3
Female	100	93	97
**Age, y, mean (range)**	40.2 (30–51)	44.1 (36–52)	42.0 (30–52)
**Race/ethnicity, %**			
Latino	100	21	70
African American	0	36	14
Asian	0	43	16
White	0	0	0
**Highest level of education completed^b^, %**			
≤8th grade	54	0	34
Some high school but did not graduate	21	7	16
High school graduate or received a GED	17	21	18
Some college or 2-year degree	4	43	18
4-year college graduate	4	21	11
≥4-year college degree	0	7	3
**Annual household income, 2009 dollars^b^, %**			
≤10,000	22	14	19
10,001-20,000	44	14	31
20,001-30,000	22	14	19
30,001-50,000	6	29	16
50,001-75,000	6	21	13
75,001-100,000	0	7	3
**Marital status^b^, %**			
Married	71	50	63
Divorced/separated	0	21	8
Widowed	4	0	3
Living with partner	13	7	11
Single	13	21	16
**Body mass index, kg/m^2^**			
Mean (range)	30.4 (23–39)	29.3 (21–45)	29.9 (21–45)
Overweight, %	39	21	31
Obese, %	44	50	47
**Self-described weight^b^, %**			
Very underweight	0	0	0
Slightly underweight	0	0	0
About the right weight	13	36	21
Slightly overweight	50	29	42
Very overweight	38	36	37
**Acculturation,^c,d^ mean (range)**	1.6 (1-2.4)	3.3 (2.2–4.4)	1.8 (1-4.4)
**Children**			
**Age, y, mean (range)**	11.9 (11–14)	12.3 (11–13)	12.1 (11–14)
**Sex, %**			
Male	57	62	58
Female	43	38	42
**Body mass index, kg/m^2^**			
Mean (range)	26.1 (13–39)	20.1 (14–31)	23.7 (13–39)
Overweight^c^, %	18	0	11
Obese^c^, %	53	18	39
**Parent’s description of child’s weight^b^, %**			
Very underweight	5	0	3
Slightly underweight	5	0	3
About the right weight	50	86	64
Slightly overweight	27	14	22
Very overweight	14	0	8

a The following data were missing: age for 7 adults; race/ethnicity for 1 adult; income for 6 adults; body mass index for 6 adults; acculturation for 1 adult; sex for 2 children; body mass index for 10 children; parent-described weight for 10 children. Values expressed as percentages unless otherwise indicated.

b Frequencies may not total 100% because of rounding.

c Overweight = 25.0 – 29.9 kg/m^2^; obese = ≥30 kg/m^2^.

d Acculturation was calculated for Latino participants only; the Short Acculturation Scale for Hispanics was used (20).

### Thematic results

Findings from the focus groups are presented in 3 thematic categories: 1) developing family-focused nutrition and physical activity programs, 2) improving access to nutrition and physical activity programs for families, and 3) identifying unmet needs of Spanish-language parents. We also identified an overarching meta-theme that permeated parents’ comments about engagement in obesity-prevention efforts. Parents were not motivated to engage in obesity prevention solely, or even primarily, to improve their children’s health but instead were interested in feeling better about themselves as parents. From this core desire flowed, almost incidentally, an interest in improving the nutrition and physical activity behaviors of their children. Parents wanted to set a good example for their children, participate in programs as a family, successfully negotiate competing family commitments, and make up for shortcomings in knowledge and skills that prevented them from maximizing their own self-satisfaction as parents.

#### Developing family-focused nutrition and physical activity programs

**Parents as role models:** Parents from both English- and Spanish-language focus groups identified the importance of parents serving as visible role models to their children regarding nutrition and physical activity behaviors. A participant in a Spanish-language focus group suggested that parents should lead by example to encourage healthy eating habits: “I think that more than anything [is] the example of the parents. If parents eat things that aren’t healthy, the children will be used to eating that. And the reverse too, if parents begin to eat healthy things, [children] will . . . too . . .”

A parent in an English-language focus group explained how parents can motivate children to exercise: “. . . if my child sees that both parents are very active, I think they are seeing that is a lifestyle. Because I think it’s a lifestyle and I think that kids have to emulate their parents. . . . I think the parent has to be the one to start exercising, and then it kind of leads into their children being active, too.”

A participant in a Spanish-language group described how her family exercises together: “In my family, all of the family likes karate or taekwondo. It was funny because a dad or a mom will arrive with their karate clothes with their [child]. . . . I always try to have them see that we are exercising.”

**Family-focused activities:** Participants in both the English- and Spanish-language focus groups believed that family-focused programs offered a viable alternative to traditional youth-only or adult-only programs. Family-focused activities allowed parents to model healthy behaviors and to use their limited time to engage in healthy activities most efficiently. Parents advocated for parent–child cooking classes and family-focused sports leagues and walking and biking clubs. A parent in a Spanish-language group expressed her desire for a parent–child nutrition program: “A nutrition class, parents and children together . . . teaching how to cook, salads, anything, together at the same time: inside the school, inside the park, anywhere.” The concept of a family-focused sports league was described by a parent in an English-language group: “I’ve actually suggested to a couple of my friends to have a parent and child softball league or something. And you could get other cities to do it, too.”

#### Improving access to nutrition and physical activity programs for families

**Staying engaged despite a busy schedule:** Time constraints were identified as a major barrier. Parents described busy work schedules and their children’s school responsibilities (eg, excessive homework, extracurricular activities) as impediments to engaging in physical activity and participating in nutrition programs.

Parents offered various suggestions to increase family involvement in community-based obesity prevention efforts. Because of busy weekday schedules, parents believed that Saturdays were best for such activities. Parents expressed the need for a local champion to encourage families to maintain participation in programs. A parent in an English-language group explained: “We cannot anticipate or expect that the same parents would be doing it over and over. There should be a resource person that . . . their job is to keep the motivation going among the parents.”

Participants also described how families could work together to encourage physical activity through group activities, such as walking clubs. A parent in one of the Spanish-language groups described her idea: “I think small communities could be made where 3 . . . 6 kids could get together from a block, they could all go for a walk with a parent. . . . There are 3 or 4 kids who live in the same block and go to school, why not make a walk where we all go together with a parent supervising them?”

**Variability in available community-based obesity prevention opportunities:** Participants’ knowledge and use of existing obesity-prevention programs in their communities varied. Some parents were aware of opportunities in their neighborhoods but reported limited use of such services. A parent in a Spanish-language group explained the need to promote local physical activity classes: “Close to my house there is a park, they have free karate, dancing, guitar classes, but unfortunately there is no participation.”

In other neighborhoods, desired programs were not available. An English-language group participant described the lack of programs nearby: “[The city] doesn’t provide hip-hop classes. I wish they do, because they get all these grants somehow but then the only community centers available that have those [classes] that the kids really like is in other communities . . . I have to travel to other cities just to take my daughter places that she likes to go.”

#### Identifying unmet needs of Spanish-language parents

**Needed skills and knowledge:** Only parents in the Spanish-language focus groups expressed a need to learn how to read nutrition labels, what constitutes a “healthy meal,” and how to identify portion sizes. A parent from a Spanish-language group explained her needs: “I would like to learn more about reading labels, because there are labels that say the calories and saturated fat, or sugars and carbohydrates and calories. That is what I don’t understand, the truth. That is one part of it. For me the nutrition is reading the labels.” Spanish-language parents also expressed a need to learn how to use a computer to navigate the Internet to learn about health issues.

**Building trust and providing culturally appropriate information:** Parents in the Spanish-language groups expressed additional frustrations with obesity-prevention programs. Some believed the facilitators were not credible or prepared. A parent in a Spanish-language focus group described the type of person she would prefer to have to facilitate a nutrition program: “. . . a person educated in nutrition — a nutritionist. Someone that can really tell you what is fine and what is not fine. A professional . . . you trust what they are saying.”

For others, issues about cultural sensitivity to Latino populations and access to Spanish-speaking facilitators were of primary concern. A parent in a Spanish-language group described her experience with obesity-prevention programs: “Los Angeles should make more [obesity] programs for Latinos . . . they talk purely English . . . the first and second part I understand, but I don’t understand the rest. Sometimes we understand [the content], but to talk it is difficult.”

## Discussion

Parents in our focus groups were interested in participating in obesity prevention programs, but their motivation to engage in such activities did not stem primarily from their desire to improve the health of their children and families. Instead, we identified an overarching desire among parents to feel better about themselves as parents that could in turn lead to pursuing improvements in their family’s nutrition and physical activity behaviors. Parents wanted to lead by example, navigate their busy lives more efficiently, and develop needed skills and knowledge that would allow them to participate in cooking classes and family sports leagues. This finding may provide support for recruiting parents to engage in obesity prevention efforts by appealing to a more general desire to become better parents. These findings were consistent among participants in both English-language and Spanish-language focus groups. To accomplish this aim, obesity prevention interventions could incorporate the development of parenting skills, such as time management and identification of teachable moments, a strategy that has been attempted with preschool-aged children (24,25).

Findings from our focus groups indicate that parents want to serve as better role models for their children regarding nutrition and physical activity behaviors. Parents acknowledged that busy schedules made it difficult for family members to engage in obesity prevention efforts. Family-focused, community-level programs emerged as a preferred approach to address obesity control, given cited barriers to participating in programs focused on the individual.

The literature on parent-involved obesity prevention is slowly emerging and suggests that parent involvement has the potential to influence children’s nutrition and physical activity behaviors (11). The level and extent to which parents are incorporated into youth obesity prevention programs vary from programs holding separate classes for parents and children and others involving parents through home-based activities (13,17,26). Results from our study confirm previous findings that parents want to be integrated and involved in obesity prevention programs (16,27). A potentially more promising extension to parent-involved interventions, however, is family-focused programming (9).

The concept of family-focused physical activity programs has emerged as a potential means to motivate parents and middle-school–aged children to exercise more regularly. Prior studies have cited family systems theories that posit that families are more likely to achieve desired health goals if they strive to reach these goals as a family unit (12,14). In our study, parents requested organized, family-focused walking, biking, or sports leagues.

The concept of parent-involved exercise programs has been mentioned in previous qualitative work (28), and the obesity prevention field has recently advocated for adopting a family-focused approach (14). Such an approach may lead to innovative interventions that address many of the issues raised by our focus groups, for example, a noncompetitive soccer league that populates teams with parents and their middle-school–aged children on Saturday mornings. Parents would have the opportunity to model engagement in physical activity, a desire they identified in our discussions. Families would be able to exercise simultaneously during a convenient hour, thus maximizing the limited time family members have to be active and together. Children and parents would have the opportunity to motivate each other and bond over their team-based experience. This parent-child bonding may in turn help facilitate an opportunity to more openly discuss other health behaviors, such as improving food choices. In addition, activities that incorporate the development of parenting skills may further encourage parents to participate. The development and evaluation of family-focused sports leagues may represent a new approach to obesity prevention and control that warrants investigation.

Less is known about engaging parents whose primary language is Spanish because they face unique challenges with accessing obesity prevention programs, including cultural appropriateness, language, and social role norms (15,16). Most parents from our Spanish-language focus groups came from Latino families who also reported low acculturation scores and identified barriers that prevent them from participating in obesity prevention efforts. Participants cited a lack of information in Spanish or programs not tailored to their cultural backgrounds, which supports previous findings (29,30). Community-based obesity prevention programs may need to develop outreach strategies that reach families whose primary language is Spanish and employ not only culturally appropriate but also credentialed, professional facilitators. Parents from the Spanish-language focus group also expressed a need to learn how to access the Internet to learn more about obesity prevention. The development of these skills would also meet their desire to become better parents. Developers of obesity prevention and control programs aimed at Latino populations may consider incorporating modules about using the Internet and developing clearinghouses of Spanish-language materials.

The use of a focus group study design imposes limitations on our findings. We selected focus group interviews to facilitate in-depth discussions of individual perspectives in the context of a group setting, which may allow individual contributions to be disproportionately represented in the aggregate group results. Our study sample, moreover, was recruited through convenience sampling and consisted of mostly Latino women, which limits the application of our findings.

Nevertheless, our findings confirm the notion that parents want to help their children avoid obesity but feel constrained in their ability to take action. Families face several barriers to healthy lifestyles at home, in schools, and throughout their communities. We discovered no major differences between parents in our English-language and Spanish-language groups related to parental motivation and interest in participating in obesity prevention programs except for language-related barriers for parents whose primary language was Spanish. Increasing the development and testing of simple programs that are sustainable, community-based, and family-focused may lead to innovations that can empower families to live healthier lives in their communities.

## Appendix. Abridged Focus Group Protocol, Assessment of Parents’ Views on Engaging Middle School Students in Obesity Prevention and Control in a Multiethnic Population

Icebreaker:

1. How much of a problem do you think unhealthy eating and physical inactivity are among boys/girls in middle school?

TOPIC 1: Nutrition and Healthy Eating

1. How did you and your family develop your concept of a healthy diet?

2. Parents have told us it can be hard for them to prepare healthy food for their children at home. What would make it easier for parents to prepare and serve healthy foods at home?

3. How can parents motivate their children to eat healthier meals and snacks?

4. What information about nutrition and healthy eating would parents like to learn more about?

5. What skills do parents need to develop to talk to their children about healthy eating?

6. What types of programs or activities for parents of middle school students would help them encourage their children to eat healthfully? Think about programs that could be provided in connection with a middle school and/or provided in a community setting.

Follow-up questions:

6a. Where should this type of program be offered?

6b. Who should provide this type of program?

7. What do you think about a program for students AND their parents? For example, a program that parents and students did together in the same session, separately, or a combination of the 2.

TOPIC 2: Physical Activity

1. How much physical activity should a middle school student engage in every day?

2. Parents have told us it can be hard to encourage their children to get enough physical activity. What would make it easier for parents to help their children engage in more physical activity?

3. What information about physical activity and exercise would parents like to learn more about?

4. What skills do parents need to develop to talk to their children about being physically active?

5. What types of programs or activities for parents of middle school students would help them encourage their children to be physically active? Think about programs that could be provided in connection with a middle school and/or provided in a community setting.

Follow-up questions:

5a. Where should this type of program be offered?

5b. Who should provide this type of program?

5c. Do you think other parents and family members would attend this type of program?

6. What do you think about a program for students AND their parents? For example, a program that parents and students did together in the same session, separately, or a combination of the 2.
